# V_2_O_5_, CeO_2_ and Their MWCNTs Nanocomposites Modified for the Removal of Kerosene from Water

**DOI:** 10.3390/nano12020189

**Published:** 2022-01-06

**Authors:** Thamer Adnan Abdullah, Tatjána Juzsakova, Rashed Taleb Rasheed, Muhammad Ali Mallah, Ali Dawood Salman, Le Phuoc Cuong, Miklós Jakab, Balázs Zsirka, Karol Kułacz, Viktor Sebestyén

**Affiliations:** 1 Sustainability Solutions Research Lab, Bio-, Environmental and Chemical Engineering Research and Development Center, Faculty of Engineering, University of Pannonia, P.O. Box 158, H-8201 Veszprem, Hungary; yuzhakova@almos.uni-pannon.hu (T.J.); ali.dawood@mk.uni-pannon.hu (A.D.S.); sebestyenv@almos.uni-pannon.hu (V.S.); 2 Chemistry Branch, Applied Sciences Department, University of Technology, Baghdad P.O. Box 19006, Iraq; 100010@uotechnology.edu.iq; 3 National Centre of Excellence in Analytical Chemistry, University of Sindh, Jamshoro 76080, Pakistan; chemist.ali.2015@gmail.com; 4 Department of Chemical and Petroleum Refining Engineering, College of Oil and Gas Engineering, Basra University, Basra P.O. Box 61004, Iraq; 5 Department of Environmental Management, Faculty of Environment, The University of Danang—University of Science and Technology, Danang 550000, Vietnam; lpcuong@dut.udn.vn; 6 Engineering Research and Development Centre, University of Pannonia, P.O. Box 158, H-8201 Veszprem, Hungary; jakab.miklos@mk.uni-pannon.hu; 7 Research Group of Analytical Chemistry, Laboratory for Surfaces and Nanostructures, Center for Natural Sciences, University of Pannonia, P.O. Box 158, H-8201 Veszprem, Hungary; zsirkab@almos.uni-pannon.hu; 8 Faculty of Chemistry, University of Wroclaw, F. Joliot-Curie 14, 50-383 Wrocław, Poland; karol.kulacz@chem.uni.wroc.pl

**Keywords:** MWCNTs, nanoparticles, metal oxide nanocomposites, kerosene removal from water

## Abstract

In this paper, the application of multiwalled carbon nanotubes (MWCNTs) based on metal oxide nanocomposites as adsorbents for the removal of hydrocarbons such as kerosene from water was investigated. Functionalized MWCNTs were obtained by chemical oxidation using concentrated sulfuric and nitric acids. V_2_O_5_, CeO_2,_ and V_2_O_5_:CeO_2_ nanocomposites were prepared using the hydrothermal method followed by deposition of these oxides over MWCNTs. Individual and mixed metal oxides, fresh MWCNTs, and metal oxide nanoparticle-doped MWCNTs using different analysis techniques were characterized. XRD, TEM, SEM, EDX, AFM, Raman, TG/DTA, and BET techniques were used to determine the structure as well as chemical and morphological properties of the newly prepared adsorbents. Fresh MWCNTs, Ce/MWCNTs, V/MWCNTs, and V:Ce/MWCNTs were applied for the removal of kerosene from a model solution of water. GC analysis indicated that high kerosene removal efficiency (85%) and adsorption capacity (4270 mg/g) after 60 min of treatment were obtained over V:Ce/MWCNTs in comparison with fresh MWCNTs, Ce/MWCNTs and V/MWCNTs. The kinetic data were analyzed using the pseudo-first order, pseudo-second order, and intra-particle diffusion rate equations.

## 1. Introduction

Environmental pollution has been a matter of concern globally from several decades. Moreover, numerous policies and frameworks have sought to deal with it. Water pollution resulting from oil spills has been comprehensively studied by researchers in order to save not only marine and aquatic life but also peoples’ lives [1]. Since water polluted with hydrocarbons has been the main reason for numerous waterborne diseases, its consumption is toxic to humans [2]. Given that mechanization has experienced exponential growth and human development primarily relies on industrial development, the exploitation of crude oil and its various derivatives for the smooth functioning of industrialization has increased. The extensive increase in terms of the exploration and refining of crude oil and its subsequent transportation worldwide has had a terrible impact on the environment. The resultant contaminated water usually contains four main types of oils: firstly, light hydrocarbons (e.g., kerosene, gasoline, etc.); secondly, heavy hydrocarbons; thirdly, lubricants and cutting fluids; and fourthly, fats derived from animals or plants [3].

Remediation techniques for these contaminants are continuously emerging in the pursuit of being more economical and efficient as well as handling a wider range of pollutants [4]. The most common methods include biological degradation, physical remediation, chemical oxidation and adsorption. Among these methods, adsorption has been most commonly used for cleaning water and remediating pollutants [5]. A long list of adsorbent materials have been applied for the purpose of remediating pollutants including modified clay minerals [6], activated carbon [7], zeolites [8], graphene [9], cellulose [10], biopolymers [11], and metal oxide nanoparticles [12,13]. However, certain problems result from the majority of these methods that limit their application as widely applicable remediation techniques. These issues include low adsorption capacity, limited recyclability, and poor selectivity [14]. The most common and most frequently used adsorbent material is activated carbon. The reason behind its restricted application is mainly attributed to two factors; its adsorption capacity and selectivity towards the removal of trace contaminants such as a certain type of heavy metal ions, is comparatively low [15,16].

Efficient environmental remediation has been witnessed over the last decade as a result of nanotechnology since nanomaterials possess manifold benefits such as large surface area, more active adsorption sites, high reactivity and small size [17,18]. Carbon nanotubes (CNTs) in comparison with other nanoparticles exhibit a relatively higher adsorption affinity towards the removal of volatile organic compounds and hydrocarbons [19] and it has highly recyclability without decrease in their efficiency [20]. This type of adsorbent material is efficiently used in water treatment for the removal of oil and toxic organic compounds [21] as well as heavy metal ions [22]. This feature is attributed to a number of characteristics of CNTs such as low density, electrical conductivity, relatively large specific surface area, high inherent strength, higher adsorption capacity, good hydrophobicity, thermal as well as chemical stability, and high aspect ratio (ratio of the length to width of a particle) [23]. Carbon nanotubes are often described as high aspect ratio nanoparticles having fast adsorption rates, appropriate oleophilic characteristics, and high hydrogen storage capacity [21]. Tan et al., has also pointed out that oil spills can seriously threaten the environment and managed to successfully develop an environmentally friendly adsorbent for the removal of spilled oil on raw corn straw after the deposition of ZnO/SiO_2_ nanocomposite particles with excellent superoleophilic and superhydrophobic characteristics [24]. Based on research, Qiming et al., reported that the titania-modified carbon nanotube composite exhibited enhanced adsorption of organic pollutants for the purpose of their removal in comparison to pure TiO_2_ nanoparticles [25]. Similarly, it was reported that functionalized silica-coated magnetic nanocomposites are efficient for the removal of organic pollutants from aqueous solutions [26]. Kirti et al., exploited the functionality of biomass in iron nanocomposites for the potential removal of four dyes, including both anionic and cationic dyes [27]. Similarly, Hassan et al., used SnO_2_/CeO_2_ nanocomposites to remove dyes [28]. Al-Jammal et al., reported a microemulsion technique for the functionalization of MWCNTs with a hydrocarbon tail to remove numerous hydrocarbons from water contaminated by spilled oil [19]. Classical methods such as diffusion, stripping, skimming, gravity separation, emulsification, chemical coagulation, dispersing, phytoremediation, or bioremediation remove emulsified oil from decontaminated water less efficiently and require high operational costs [29]. Therefore, researchers prefer to combine methods [30]. Detailed analysis of the relevant literature showed that adsorption is a promising method being highly efficient, cost-effective and environmentally friendly [31,32]. Similarly, the modification of MWCNTs results in an increase in the hydrophobicity with regard to adsorption and subsequent removal of hydrocarbons from oil spills [33]. Usually, the modified surface structure exhibits improved properties concerning hydrophobicity and hydrophilicity, which is very important for the separation of oil-in-water emulsions [19,24]. 

This research aimed to develop advanced adsorbents for the removal of hydrocarbons from surface water via chemical oxidation of MWCNTs followed by doping the MWCNTs with nanometal oxides (V_2_O_5_, CeO_2_, and V_2_O_5_:CeO_2_). Modifying the metal oxides decorates the MWCNTs with more functional/adsorption groups. In this work, the structural and surface chemistry properties of the prepared adsorbents are studied by different surface analytical techniques and correlated with their adsorption efficiency with regard to the removal of kerosene from a model aqueous solution. Adsorption kinetic models will be applied to study the adsorption behavior of the fresh MWCNTs and metal oxide nanoparticle-doped MWCNTs with regard to the removal of kerosene.

## 2. Materials and Methods

### 2.1. Materials and Methods

The following chemicals were used: ammonium metavanadate (NH_4_VO_3_, 99.99%), nitric acid (HNO_3_, 99%), cetrimonium bromide (CTAB, C_19_H_42_BrN, 99%), ethanol (CH_3_CH_2_OH, 99.8%), cerium(IV) sulfate tetrahydrate (Ce(SO_4_)_2_·4H_2_O, 99.0%) and urea (NH_2_CONH_2_, 99%) were purchased from Merck Kft., Budapest, Hungary as well as hydrochloric acid (HCl, 99.7%), sodium hydroxide (NaOH, 99.0%) and sulfuric acid (H_2_SO_4_, 99.7%) were purchased from VWR International Kft., Debrecen, Hungary. Commercial MWCNTs (TNNF-6 type, Timesnano, Chengdu, China) made by chemical vapor deposition (CVD) were used in this research.

### 2.2. Preparation of Metal Oxide Nanoparticles

#### 2.2.1. Preparation of Vanadium Oxide Nanoparticles

Vanadium pentoxide (V_2_O_5_) was prepared by hydrothermal methods (reflux) using ammonium metavanadate, ethanol (EtOH), nitric acid, cetyltrimethylammonium bromide (CTAB), and distilled water according to the reported method [34,35]. At first, 0.1 g of NH_4_VO_5_ and 0.1 g of CTAB were dissolved in a mixture of distilled water and ethanol (100 mL) in the ratio of 7:3, respectively. This was followed by adding HNO_3_ very slowly whilst continuously being stirred until the pH value became 2.5. The mixture above was heated under reflux for 6 h at 80 °C. The precipitate was washed with distilled water 10 times before being washed with ethanol. Then it was dried in an oven at 90 °C for 60 min and annealed at 500 °C for 4 h to obtain the vanadia nanoparticles. Equation (1) describes the chemical reactions used to prepare vanadium pentoxide [34].

(1) $$2{\mathrm{NH}}_{4}{\mathrm{VO}}_{3}+2{\mathrm{HNO}}_{3}+{\;\mathrm{nH}}_{2}O\;\overset{\mathrm{EtOH}/\mathrm{CTAB}}{\to }V_{2}O_{5}{\mathrm{nH}}_{2}O+2{\mathrm{NH}}_{4}{\mathrm{NO}}_{3}+H_{2}O$$

#### 2.2.2. Preparation of Cerium Oxide Nanoparticles

Cerium dioxide (CeO_2_) was prepared by a hydrothermal method using an autoclave as well as ceric sulfate tetrahydrate, urea, CTAB and distilled water. At first, 2.34 g of Ce(SO_4_)_2_·4H_2_O, 1.39 g of NH_2_CONH_2_ and 2.1 g of CTAB were dissolved in 50 mL of distilled water before being mixed on a magnetic stirrer for 30 min. The materials were put into a 50 mL autoclave and kept at 200 °C for 12 h. Following this, the autoclave was left to cool down to room temperature. The Ce(OH)_4_ obtained was washed with distilled water several times before being centrifuged and then washed again with ethanol. The sample was dried at 90 °C for 60 min and then annealed at 500 °C for 4 h. Equations (2)–(4) describe the chemical reactions used to prepare cerium dioxide:

(2) $${\mathrm{NH}}_{2}{\mathrm{CONH}}_{2}+3H_{2}O\;\to \;2{\mathrm{NH}}_{4}\mathrm{OH}+{\;\mathrm{CO}}_{2}$$

(3) $$\mathrm{Ce}{\left({\mathrm{SO}}_{4}\right)}_{2}\cdot 4H_{2}O+4{\mathrm{NH}}_{4}\mathrm{OH}\;\to \mathrm{Ce}{\left(\mathrm{OH}\right)}_{4}+2{\left({\mathrm{NH}}_{4}\right)}_{2}{\mathrm{SO}}_{4}+4H_{2}O$$

Annealing at 500 °C yields:

(4) $$\mathrm{Ce}{\left(\mathrm{OH}\right)}_{4}\to {\;\mathrm{CeO}}_{2}+2H_{2}O$$

#### 2.2.3. Preparation of Mixed V_2_O_5_:CeO_2_ Nanocomposites

The composite V_2_O_5_:CeO_2_ was prepared by mixing V_2_O_5_ and CeO_2_ in the mole ratio of 3:1, respectively. Metal oxides were mixed in a beaker with ethanol for 6 h on a magnetic stirrer before the mixture was placed into a furnace and annealed for 2 h at 500 °C.

### 2.3. Functionalization of MWCNTs with Metal Oxide Composites

MWCNTs were functionalized using the concentrated acids H_2_SO_4_ and HNO_3_ in a ratio of 3:1, respectively. The process of modification was carried out using the strong oxidizing agents HNO_3_ and H_2_SO_4_ to introduce carboxylate groups onto the MWCNTs. The mixture of concentrated acids was put into a beaker and 2 g of MWCNTs was added before being ultrasonicated for 30 min. Following this, the mixture was transferred to a round-bottom flask to be heated under reflux for 8 h at 180 °C before being diluted 15 times with distilled water, filtered by a membrane filter and washed until the pH became 7. The addition of functionality onto the surface of the nanotubes was carried out in our research by metal oxides and their composite nanoparticles. 2 wt% of the prepared metal oxides CeO_2_ and V_2_O_5_ as well as their mixed nanocomposites, respectively, were added to MWCNTs in 70 mL of ethanol. The solution was stirred at 40 °C for 4 h using a magnetic stirrer and ultrasonicated for 30 min before being heated under reflux for 4 h at 90 °C. The solution was transferred to an autoclave reactor and kept at 200 °C for 4 h. Finally, the samples were dried by evaporating off the ethanol at 85 °C.

### 2.4. Characterization Methods

The specific surface area, pore volume, and pore-size distribution within the ranges of diameters of micropores (1.7–2 nm), mesopores (2–50 nm), and macropores (50–100 nm) were determined by nitrogen adsorption/desorption isotherms at −196 °C using an analyzer ASAP 2000 manufactured by Micromeritics, Norcross, GA, USA. The specific surface areas of the samples were determined by the BET (Brunauer–Emmett–Teller) method from the corresponding nitrogen adsorption isotherms. The pore-size distribution and pore volumes were calculated from the nitrogen desorption isotherms using the BJH (Barret–Joyner–Halenda) model.

Identification of the solid crystalline phases in the samples was determined by X-ray diffraction analysis (XRD) using a Philips PW3710 X-ray diffractometer equipped with Cu-Kα radiation (λ = 0.1541 nm) and recorded at room temperature over the 4–70° 2θ angular range with a scanning acquisition speed of 0.02 °/s.

The morphology of the surface of the nanocomposites was studied using the transmission electron microscopy (TEM) and scanning electron microscopy (SEM) techniques. The nanocomposites for TEM were prepared by depositing a drop of nanocomposites suspended in ethanol on copper grids covered by an amorphous lacey carbon support film. SEM and TEM analyses were performed using a Thermo Fisher Scientific Apreo S LoVac SEM in the Czech Republic operated at 2.0 kV for backscattered electron imaging and at 30.0 kV for transmission electron imaging, equipped with an energy-dispersive X-ray spectrometer (EDX), (AMETEK’s Octane Elect Plus, Berwyn, PA, USA).

Atomic Force Microscopy (AFM) analysis were carried out using a SPM-AA 3000 type instrument. AFM was used to probe the sample surface in nanometer scale in order to investigate the surface roughness and surface particle-size distribution. The samples were dissolved in ethanol and transferred onto a glass plate of 0.5 mm × 0.5 mm size for analysis.

Raman spectra were recorded using a Bruker RFS 100/S FT–Raman spectrometer with a Nd:YAG laser source (1064 nm, 30 mW) and a liquid N_2_ cooled Ge detector. Signal-to-noise ratio was improved by the coaddition of 2048 spectra with a resolution of 4 cm^−1^. Spectral deconvolution of the baseline corrected Raman spectra were achieved by fitting a mixture of Gaussian and Lorentzian line shapes in PeakFit software (v4.12, Seasolve, Systat Software, San Jose, CA, USA). Thermoanalytical measurements (TG/DTG) were carried out using a Netzsch TG-209 type thermobalance. Samples were measured in ceramic crucibles. The TG/DTG curves were registered while heating the samples to 1000 °C (10 °C/min heating rate) in dynamic argon flow (99.998%).

### 2.5. Adsorption Test

The adsorption tests over fresh MWCNTs, Ce/MWCNTs, V/MWCNTs, and V:Ce/MWCNTs nanosorbents were carried out with kerosene cut to study the efficiencies with regard to the removal of hydrocarbons from samples of water. Adsorption experiments were carried out in a batch mode at room temperature. The model contaminated stock solution was made in a laboratory using a kerosene concentration of 500 mg/L in distilled water. The 50 mL of model solutions were mixed for 20 min using a magnetic stirrer before adding 5 mg of the adsorbent.

Then each solution was shaken for between 15 and 60 min at room temperature. The adsorbent was separated from the solution by filtration. The filtered aqueous solution was taken for the extraction step to prepare the sample for gas chromatography (GC) in order to determine its hydrocarbon content. The kerosene-water solution was extracted with 20 mL of hexane over 30 min and shaking at a rotational speed of 240 rpm. The hexane fraction was dried with Na_2_SO_4_ powder. The blank solution was prepared by the same method without adding any adsorbent. The kerosene-hexane samples were analyzed by an Agilent 7890A Gas Chromatograph with a J and W HP-5 capillary column (30 m × 0.320 mm, 0.25 µm film thickness). A flame ionization detector (FID) was used for the analysis.

The efficiency of kerosene removal (*RE*%) and the quantity of kerosene adsorbed (*q_t_*) were calculated by Equations (5) and (6), respectively [19,36]:

(5) $$RE=\left(\frac{C_{0}-C_{t}}{C_{0}}\right)\cdot 100\%$$

(6) $$q_{t}=\frac{\left(C_{0}-C_{t}\right)}{m}\times V$$

where *C*_0_ denotes the initial kerosene concentration, mg/L; *C_t_* stands for the final kerosene concentration at time *t*, mg/L; *q_t_* represents the adsorption capacity at time *t*, mg/g; *V* refers to the volume of kerosene solution, L; and *m* is the weight of adsorbent, g.

### 2.6. Kinetic Studies

In order to investigate the mechanism of kerosene sorption on metal oxide-modified MWCNTs surface and examine the potential rate-controlling step, the pseudo-first order, pseudo-second order, and intra-particle diffusion kinetic models were studied.

## 3. Results

### 3.1. X-ray Diffraction

**Figure 1:**
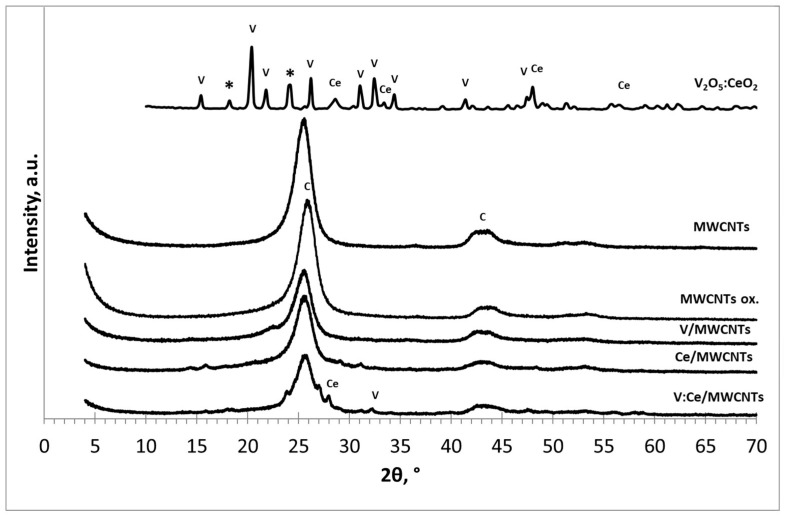
XRD results for fresh and modified MWCNTs, where V, Ce, and C denote the V_2_O_5_, CeO_2_ and graphite crystalline phases and * represents impurities.

Figure 1 shows the XRD results for the samples of V_2_O_5_:CeO_2_ fresh MWCMTs, oxidized MWCNTs, V/MWCNTs, Ce/MWCNTs, and V:Ce/MWCNTs. V_2_O_5_:CeO_2_ nanoparticles prepared by the hydrothermal method and annealed at 500 °C showed reflections of both oxides, namely V_2_O_5_ and CeO_2_. In the case of vanadia nanoparticles, the main diffraction peaks of (200), (010), (110), (101), (310), (011), (301), (020) and (320) appear at 15.04°, 20.04°, 21.80°, 26.20°, 31.00°, 32.40°, 34.20°, 41.20° and 48.00° 2θ, respectively. These peaks relate to the shcherbinaite orthorhombic crystalline structure of vanadium pentoxide (JCPDS Card No. 41-1426) [34]. The main diffraction peaks of CeO_2_ are (111), (200), (220) and (311) at 28.60°, 32.20°, 47.40° and 56.51° 2θ, respectively, in the form of cerianite, as indexed in JCPDS Card No. 34-0394 [37]. In mixed composites, the constituent V_2_O_5_ orthorhombic crystalline structure dominates over the CeO_2_ equivalent. Furthermore, the presence of some impurities was observed at 18.20° and 24.20° 2θ originating from the preparation step [38].

The oxidized MWCNTs, V/MWCNTs, Ce/MWCNTs, and V:Ce/MWCNTs samples were treated at 200 °C. On the basis of the XRD results, it can be seen that the graphene layers of MWCNTs are preserved after acid treatment and deposition of metal oxides. The diffraction peaks at 25.3° and ~43° 2θ of metal oxide-modified MWCNTs are (002), (100) and (101) reflections of graphite, as indexed in JCPDS Card No. 01-071-4630 [39]. The peaks corresponding to CeO_2_ and V_2_O_5_ in Ce/MWCNTs and V/MWCNTs could not be detected possibly due to their nominal addition. The major crystalline phase with regard to the graphene layers of the MWCNTs was recorded for all multiwalled carbon nanotubes. The presence of some crystals of individual oxides was detected for samples of V:Ce/MWCNTs.

### 3.2. Electron Spectroscopy and Energy Dispersive X-ray *Spectroscopy* Results

**Figure 2:**
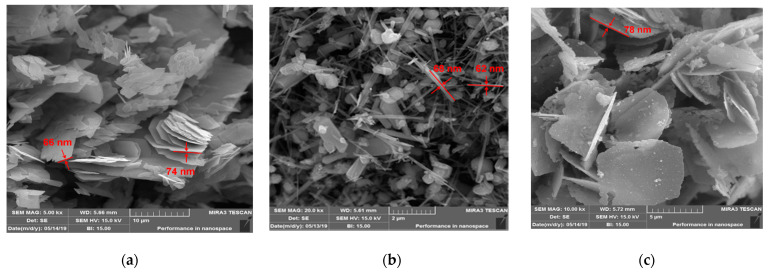
SEM images between 2 and 10 µm: (**a**) V_2_O_5_, (**b**) CeO_2_, (**c**) V_2_O_5_:CeO_2_.

**Figure 3:**
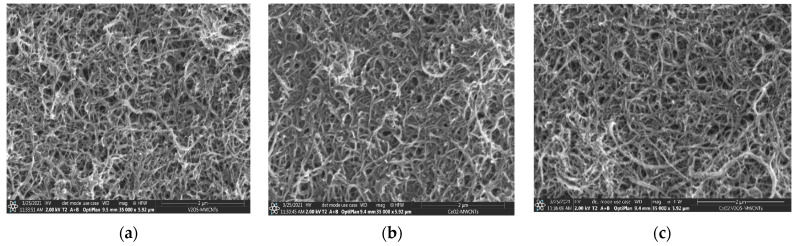
SEM images on scale of 2 µm: (**a**) V/MWCNTs, (**b**) Ce/MWCNTs, (**c**) V:Ce/MWCNTs.

**Figure 4:**
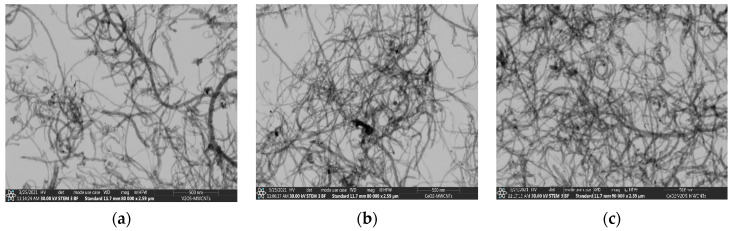
TEM images on a scale of 500 nm: (**a**) V/MWCNTs, (**b**) Ce/MWCNTs, (**c**) V:Ce/MWCNTs.

SEM records for metal oxides are shown in Figure 2a–c. Figure 2a shows the surface morphology of V_2_O_5_ nanopowder, identified in the form of nanoflakes close to 65–80 nm in thickness, which is in agreement with the atomic force microscopy (AFM) results. Figure 2b shows that the surface morphology of the CeO_2_ powder adopts a different shape including nanorods, the diameter of which can be seen on the nanoscale (60–70 nm) and is also shown by the AFM analysis. Figure 2c shows the V_2_O_5_:CeO_2_ nanocomposite in which the vanadium pentoxide is preserved in the form of nanoflakes. SEM and TEM records for fresh MWCNTs and for metal oxide-modified MWCNTs are shown in Figure 3 and Figure 4. It can be observed that the size of the carbon nanotubes remains within the nanoscale range and the shape of carbon nanotubes was not significantly affected during thermal modification of MWCNTs in the presence of nanoparticles (Figure 4a–c).

EDX results were recorded to determine the amount of carbon, vanadium and/or cerium on the surface of the samples. The analysis was carried out on three areas of the specimens and the average measured compositions of the near-surface layer of the samples are presented in Table 1. The compositions of the V/MWCNTs and V:Ce/MWCNTs samples estimated by EDX analysis were in good agreement with the theoretical values used for preparation. Higher amounts of cerium dioxide than expected were detected in the Ce/MWCNTs samples. The EDX results support the notion that metal oxides were deposited on the surface of the carbon nanotubes.

### 3.3. Atomic Force Microscopy Results

**Figure 5:**
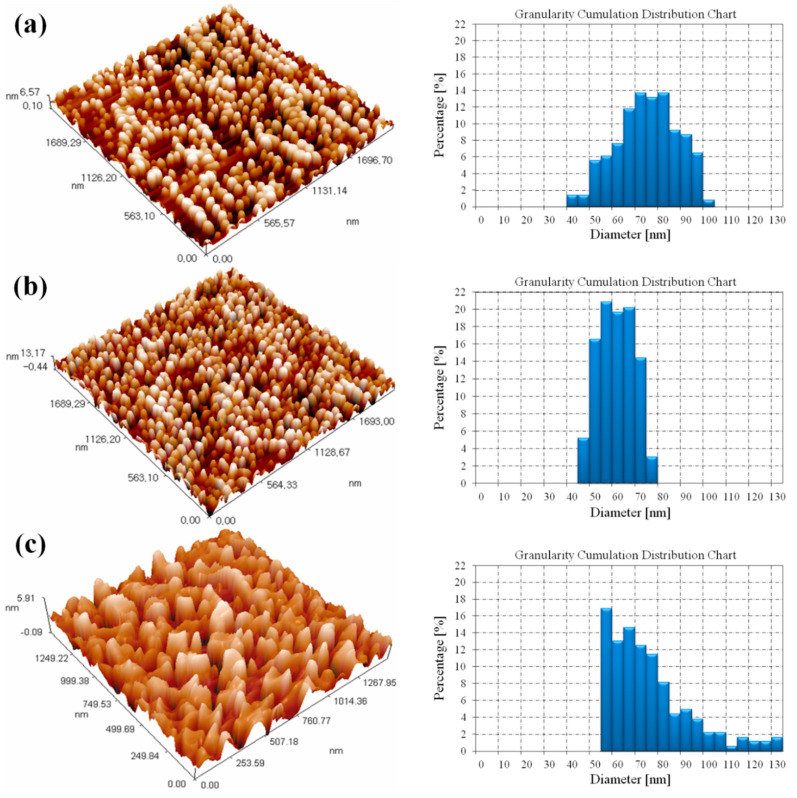
AFM records of (**a**) V_2_O_5_, (**b**) CeO_2_, and (**c**) V_2_O_5_:CeO_2_ composites at an annealing temperature of 500 °C.

AFM in three-dimensional images of V_2_O_5_, CeO_2_ and V_2_O_5_:CeO_2_ nanoparticles after annealing at 500 °C are shown in Figure 5. The morphological images of V_2_O_5_, CeO_2_ and V_2_O_5_:CeO_2_ nanoparticles clearly depict that the average grain sizes were found to be 70.3, 56.9, and 71.4 nm as shown in Figure 5a–c, respectively. The addition of cerium dioxide to vanadium pentoxide resulted in a slight increase in the particles size distribution range from 40–100 nm to 55–120 nm.

### 3.4. Raman Spectroscopy Results

**Figure 6:**
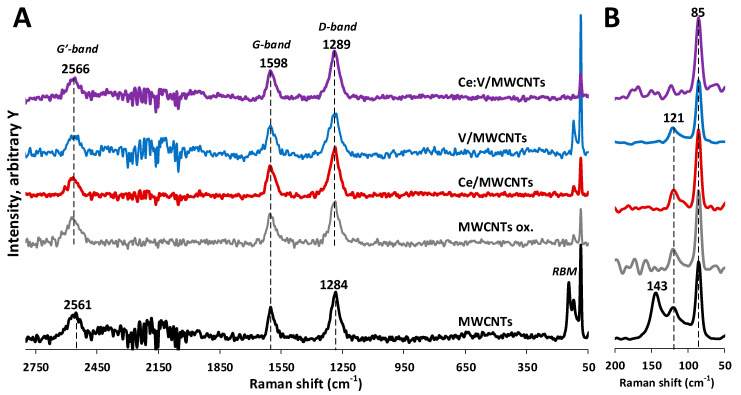
Raman spectra of the MWCNTs samples: (**A**) normalized to the D-band intensity in the 2750–50 cm^−1^ region, (**B**) normalized to the RBM bands in the 200–50 cm^−1^ region.

The distinctive features of CNTs can be identified by Raman spectroscopy (Figure 6 MWCNTs). The radial breathing mode (RBM) corresponds to the in-phase, radial movement of the carbon atoms within the structure of CNTs. It is usually not present in the Raman spectra of MWCNTs due to the weakening effect of their larger diameters [40,41]. However, two separate RBM bands were observed at 121 and 143 cm^−1^. Since the position of an RBM band is sensitive to the diameter of the CNTs, it can be utilized to estimate their overall average diameter [40,42]. The 121 and 143 cm^−1^ bands indicate the presence of two small, slightly different diameters with averages of 2.1 and 1.8 nm, respectively. The band at 1284 cm^−1^ can be assigned to the presence of defect sites in the sp^2^ carbon structure (D-band). The position of the D-band depends on the diameter and chirality of the carbon structure as well as applied laser wavelength [42]. The D-band can be deconvoluted into two bands centered on the same wavenumber for small crystallites of CNTs [43]. The peak at 1598 cm^−1^ is attributed to the vibrations of the sp^2^ carbon structure (G-band). Splitting of the G-band (G^+^-G^−^) is not observed, as is expected for MWCNTs [44]. The band at 2561 cm^−1^ (G’-band) is the overtone of the D-band, but contrary to the D-band, it indicates the long-range order of the structure arising from defect-free sp^2^ carbon atoms [40,41].

As a result of being treated with strong acids, structural defect sites are introduced in the form of carboxyl, carbonyl, and hydroxyl groups via oxidation of the carbon structure [44,45]. After oxidative treatment, the RBM band at 143 cm^−1^ disappeared. Moreover, the D- and G’-bands shifted slightly to higher wavenumbers (1289 and 2566 cm^−1^, respectively), indicating the successful surface modification (Figure 6 MWCNTs ox.). The peak intensity or area ratios of the D- and G-bands $\left(\frac{I_{D}}{I_{G}},\frac{A_{D}}{A_{G}}\right)$ can be used to estimate the quality of the CNT structure and the extent of surface modification via the presence of defect sites [42,43]. Alternatively, the G’- and D-band ratios $\left(\frac{I_{G^{\prime }}}{I_{D}},\frac{A_{G^{\prime }}}{A_{D}}\right)$ can be used for a more accurate estimation [46]. The calculated intensity ratios follow a similar trend to the area ratios (Table S1). After oxidative treatment, the $\frac{I_{D}}{I_{G}},\;\frac{A_{D}}{A_{G}}$ values decreased, while the $\frac{I_{G\prime }}{I_{D}},\frac{A_{G\prime }}{A_{D}}$ values increased (Table S1), indicating a slightly more disordered structure as a result of acid-treatment [47]. No major spectral changes were observed once the nanocomposites had been prepared (Table S1, Figure 6 Ce/,V/, V:Ce/MWCNTs). Most probably, due to their low surface concentration, no new peaks were identified in accordance with the Raman scattering of V_2_O_5_ [48] or CeO_2_ [49] polymorphs. However, based on their D- and G’-band ratios, the structural order of the V and Ce nanocomposites surprisingly showed a slight increase, compared to the MWCNTs (Table S1).

### 3.5. Thermogravimetric Analysis Results

**Figure 7:**
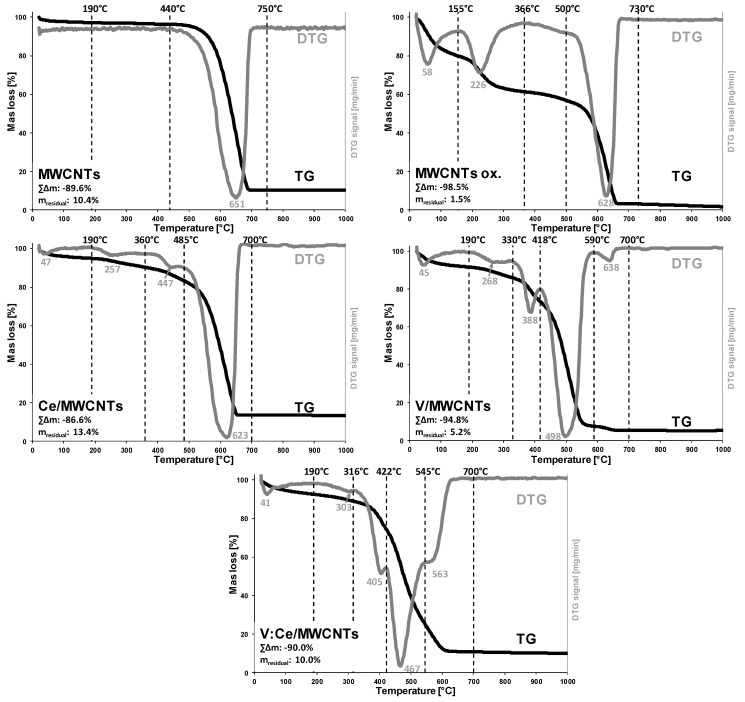
Thermogravimetric curves (TG/DTG) of the MWCNTs samples.

The untreated MWCNTs sample used for surface modification shows two notable regions (Figure 7 MWCNTs). A minor mass loss is observed up to 190 °C as the surface adsorbed water is removed, while a major mass loss step is found between 440 and 750 °C, indicating the thermal decomposition of the carbon structure (Table S2 MWCNTs). After annealing to 1000 °C the residual mass indicates the impurity of the MWCNTs sample (m = 10.4%), which is due to the presence of catalyst residues from the production of CNTs. The observed amount of catalyst impurity is well within the acceptable range reported in the literature (cc. 6–43%) [46,50].

As a result of surface treatment using strong acids, the MWCNTs structure is oxidized via the introduction of oxygenated-surface functional groups (carboxyl, carbonyl, hydroxyl) [51]. The improved hydrophilic nature due to the modified surface-properties of the acid treated MWCNTs sample is indicated by the increased amount of adsorbed water (Figure 7 MWCNTs ox., Table S2 MWCNTs ox., 21–155 °C). Oxidation of the carbon backbone results in the presence of defect sites eventuating in the lower thermal stability of the functionalized CNTs [52]. The elimination of the introduced surface carboxyl groups is observed in the second step (155–366 °C), while a small mass loss between 366 and 500 °C could be attributed to the decomposition of other, thermally more stable surface functional groups [51]. The major decomposition was found between 500 and 730 °C. As compared to the pristine MWCNTs, the shift to higher starting temperature and the narrower decomposition curve indicates the purification effect of acid treatment, mostly via the removal of carbon impurities. The significantly smaller residual mass (m = 1.5%) is due to the removal of catalyst residuals during acid treatment [51]. Consequently, the acid treatment resulted in a purified MWCNT sample.

Small changes are observed upon the addition of CeO_2_ to the oxidized MWCNTs (Figure 7 Ce/MWCNTs, Table S2 Ce/MWCNTs). No thermal decomposition process is expected due to the presence of pre-annealed CeO_2_ particles [53]. Therefore, the differences could be related to the interactions of the lanthanide-oxide nanoparticles and the carbon nanostructure. The slightly higher temperature maxima (257 °C vs. 226 °C) of the second stage could indicate a possible interaction between the CNTs surface carboxyl groups and the introduced CeO_2_ resulting in the increased thermal stability of the surface moieties. The thermal stability of the MWCNTs backbone has not changed considerably (628 °C vs. 623 °C). However, the increased mass loss of the third stage (360–485 °C) could be indicative of the increased ratio of MWCNT particles having slightly smaller thermal stability. This effect could be related to the thermal activation of CeO_2_ catalyst at elevated temperatures, resulting in a slightly enhanced pyrolysis of CNTs [54,55].

Similar changes can be observed in case of V_2_O_5_ nanoparticle addition (Figure 7 V/MWCNTs, Table S2 V/MWCNTs). No additional mass loss is expected due the addition of previously heat treated V_2_O_5_ particles [52]. The thermal stability of the second stage increased further (T_max_ = 268 °C), indicating the slightly stronger interaction of vanadium-pentoxide particles with the functionalized CNTs surface. Compared to the MWCNTs ox. sample, major changes can be observed in the thermal stability of the CNTs backbone: the mass loss ratio of the third stage has increased and the maxima has shifted to 388 °C, while the prominent decomposition stage has also shifted to 498 °C from 628 °C. Only a small mass loss at 638 °C maxima indicates the presence of MWCNTs particles with their original thermal stability. These changes can be indicative of the thermally activated catalytic properties of V_2_O_5_ nanoparticles [56], resulting in enhanced pyrolysis and lower thermal stability of MWCNTs.

The joint presence of CeO_2_ and V_2_O_5_ particles resulted in the further destabilization of MWCNTs thermal stability (Figure 7 V:Ce/MWCNTs, Table S2. V:Ce/MWCNTs). Thermal decomposition of the MWCNTs backbone slightly shifted to the lower temperature regions, where three overlapping stages can be identified (316–422 °C, 422–545 °C, 545–700 °C). The observed shift to lower thermal stability can be explained by the increased catalytic activity due to the simultaneous presence of CeO_2_ and V_2_O_5_ catalysts [55]. Overall, the addition of V_2_O_5_ particles significantly decreased the thermal stability of MWCNTs. The residual masses after 1000 °C heating can be utilized to estimate the metal-oxide content of the samples (Table S2). Considering the residual mass of MWCNTs ox. sample (m = 1.5%), the estimated oxide contents for the Ce/, V/ and V:Ce/MWCNTs samples are 11.9%, 3.7% and 8.5%, respectively, which is slightly greater than the values obtained by EDX (Table 1). The difference can be explained by the higher uncertainty of EDX, since the analytical signal originates from a smaller volume (in a scale of μm^3^) and conclusions to the overall composition bears greater errors due to the possible inhomogeneity of the sample [47]. Overall, the metal-oxide content of the MWCNTs composites are found to be greater than their theoretical values.

### 3.6. Low-Temperature Nitrogen Adsorption

The surface area and pore volume, as well as the average pore sizes of fresh, acid-treated, and metal oxide-modified MWCNTs samples, are presented in Table 2. The total surface area of fresh MWCNTs was 156 m^2^/g, which is higher than reported by the supplier (120 m^2^/g). Newly prepared samples with masses of 0.5–1.0 g previously outgassed in a vacuum at 160 °C were used for the nitrogen adsorption experiments. The mass of the sample was reduced during the outgassing procedure before the BET analysis. The highest reduction in mass was observed in the case of oxidized MWCNTs, 7.9 wt% to be exact (Table 2). It can be assumed that some moisture, namely the acid condensate, remained in the bulk of the sample after the acid treatment which was followed by outgassing and pretreatment of the sample at 100 °C before nitrogen adsorption analysis.

**Figure 8:**
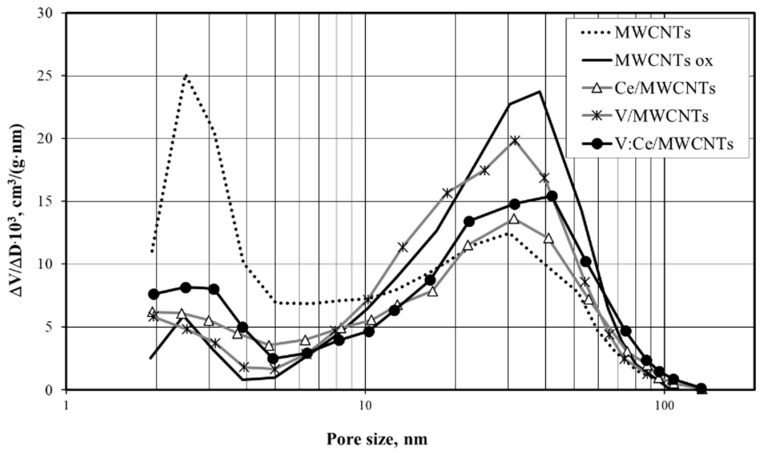
Pore volume distribution of samples of fresh, oxidized MWCNTs and metal oxide-modified MWCNTs.

The result of the treatment of MWCNTs with a 3H_2_SO_4_:1HNO_3_ mixture could be due to defect sites formed on its surface resulting in splitting of the tubes [57]. This can create new openings in the channels of the tubes, thereby increasing their total volume (Table 2). Meanwhile, the acid treatment applied resulted in a significant decrease in the micropore volume of MWCNTs ox. The acid treatment of MWCNTs could form carboxyl and hydroxyl functional groups which might block the small pore openings [58]. This can also have an impact on the total pore size distribution (Figure 8) as well as result in a slight decrease in S_BET_ from 156 to 140 m^2^/g.

CeO_2_ shows a relatively higher surface area (69 m^2^/g) among the metal oxides. The pore volume and surface area of the micropores of CeO_2_ nanoparticles exhibit higher values than those of V_2_O_5_ and CeO_2_:V_2_O_5_. V_2_O_5_ has the lowest surface area of 3 m^2^/g and the CeO_2_:V_2_O_5_ nanocomposite has a surface area of 9 m^2^/g as shown in Table 2. The surface area of mixed metal oxides depends on the proportions of the individual oxides and on the interactions between the constituent oxides. The surface area is an important factor in the studied adsorption steps.

As a result of the deposition of metal oxides over the oxidized MWCNTs, the pore volumes and pore diameters of the metal oxide-modified samples were decreased (Table 2) in comparison with acid-treated MWCNTs. In addition to this, it was also observed that the micropores were completely blocked and disappeared after the deposition of CeO_2_ or CeO_2_:V_2_O_5_ as shown in Table 2. In the case of the addition of V_2_O_5_ to MWCNTs, the surface area of the micropores decreased by 50%. The pore size distributions of these preparations are shown in Figure 8. Two ranges of pore size, namely 2–3 and 20–40 nm, were identified for all the samples. The number of smaller pores (2–3 nm) decreases and that of the larger pores (20–40 nm) slightly increases during the acid treatment of MWCNTs and in the case of metal oxide-modified MWCNTs samples.

### 3.7. Kerosene Adsorption from Water Samples

**Figure 9:**
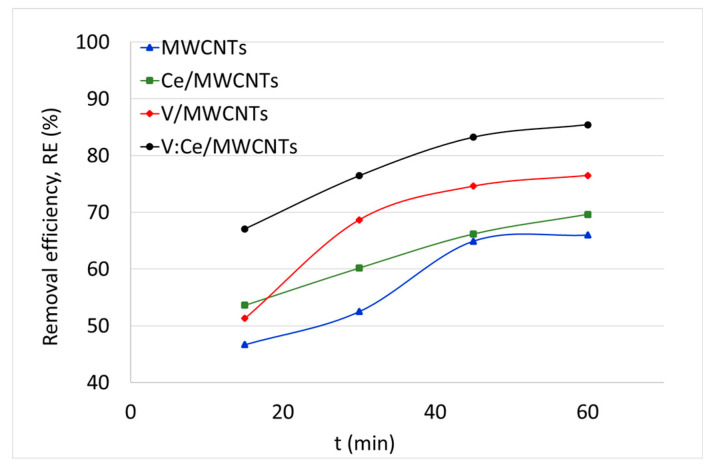
Removal efficiency of kerosene from water against time over MWCNTs, Ce/MWCNTs, V/MWCNTs, and V:Ce/MWCNTs (*C*_0_ = 500 mg, V_sample_ = 0.05 L, m_ads_ = 0.005 g).

The adsorption of kerosene from water by fresh MWCNTs and metal oxide-modified MWCNTs was studied using the GC method and the results are summarized in Figure 9 and Table 3 as well as Figure S1 in supplementary part. The initial kerosene concentration in water was 500 mg/L and the weight of each sample was 5 mg. The effect of the adsorption time on the kerosene concentration and removal efficiency was studied at different times between 15 and 60 min over the metal oxide-modified MWCNTs samples. The kerosene concentration reached its lowest value 60 min after being pretreated with all the prepared adsorbents (Figure S1). Therefore, the removal efficiency of kerosene was higher after 60 min for all samples as shown in Figure 9. The removal efficiency of samples increased rapidly up until 45 min, after which it slowly decreased for the following 15 min. GC results in Table 3 show that the preparation of V:Ce/MWCNTs exhibited the highest removal capacity and efficiency of kerosene from water (*q_t_* = 4271 mg/g, *RE* = 85%) after an adsorption time of 60 min regarding the V/MWCNTs (*q_t_* = 3825 mg/g, *RE* = 77%), Ce/MWCNTs (*q_t_* = 3481 mg/g, *RE* = 70%) and fresh MWCNTs (*q_t_* = 3300 mg/g, *RE* = 66%).

### 3.8. Kinetic Studies

The interactions between the sorbents and adsorbents are explained by a few theoretical approaches such as equilibrium isotherms and adsorption kinetics. Adsorption equilibria explain the physicochemical processes involved in sorption and kinetic measures [32,59,60]. It also explains the degree of the transport mechanism of wastewater pollutants into the adsorbent which is comprised of the external mass transfer of the sorbate from the bulk solution to the surface of the sorbent, the internal diffusion of the sorbate to the adsorption site, and the overall adsorption process [33,61]. The kinetic models are relatively efficient when determining the rate at which the adsorbent efficiently removes the adsorbate such as kerosene.

**Figure 10:**
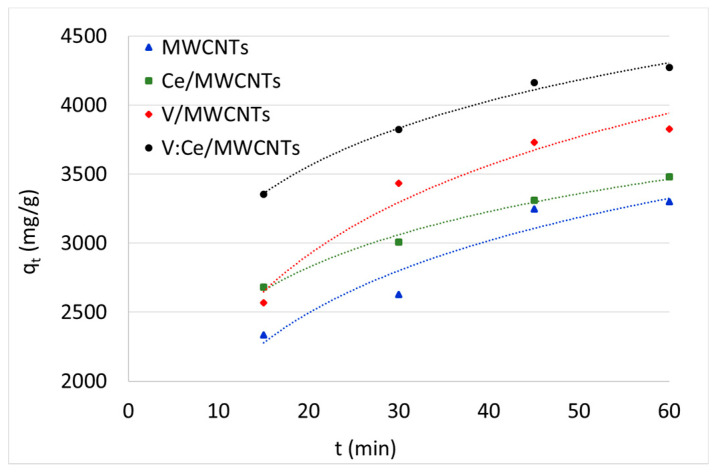
Adsorption capacity of kerosene against the time over fresh and modified MWCNTs.

Figure 10 shows the changes in kerosene amount adsorbed (*q_t_*) by samples as a function of time. Approximately, half of the hydrocarbon amount (250 mg/L) is removed from water by all samples within the first 15 min of treatment (Figure S1). Further adsorbed quantity rises more slowly as the surface become saturated with the adsorbate. Similar adsorption behavior was reported for nitrate [62] and dye molecules removal [63].

The adsorption capacity study shows that fresh MWCNTs has lower *q_t_* values than the metal oxide modified MWCNTs samples due to the availability of more active sorption sites.

In order to ascertain the reproducible results, three different kinetic models (pseudo-first order, pseudo-second order and intra-particle) were applied to study the adsorption kinetics of kerosene on fresh MWCNTs, Ce/MWCNTs, V/MWCNTs, and V:Ce/MWCNTs nanocomposites.

The pseudo-first order reaction rate constant was calculated by Equation (7):

(7) $$\log\left(q_{e}-q_{t}\right)=\log q_{e}-k_{1}\frac{t}{2.303}\;$$

where *q_e_* and *q_t_* denote the amount of kerosene adsorbed (mg/g) at equilibrium and at time t (min), respectively, while k_1_ stands for the first-order rate constant (min^−1^).

The gradient and intercept of the plots of log (*q_e_* − *q_t_*) versus t were used to determine the *k_1_* and *q_e_*, which are recorded in Table 4. From Figure S2 in supplementary part, it can be seen that the pseudo-first order reaction appropriately fitted to the experimental data for metal oxide-modified MWCNTs (R^2^ ~ 0.90 – 0.97). The value of *q_e cal_* for V:Ce/MWCNTs was closer to the experimental values obtained.

**Figure 11:**
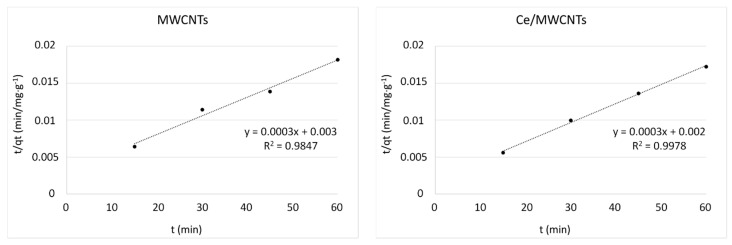

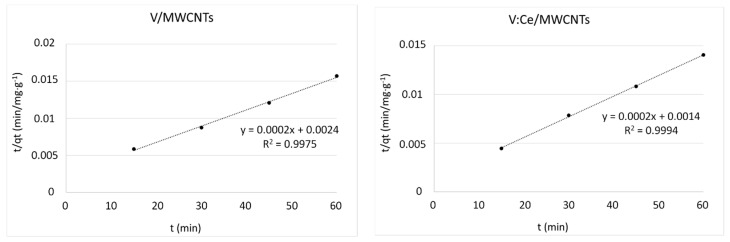
Pseudo-second order plot for kerosene adsorption onto metal oxide-modified MWCNTs.

A pseudo-second order reaction based on the equilibrium capacity of adsorption is given by Equation (8) [64]:

(8) $$\frac{t}{q_{t}}=\frac{1}{k_{2}q_{e}^{2}}+\frac{t}{q_{e}}\;$$

where *k*_2_ denotes the equilibrium rate constant of pseudo-second order adsorption (g/mg min). The values of *q_e_* and *k*_2_ were calculated from the gradient and intercept of the linear plot of *t*/*q_t_* against *t* (Figure 11), respectively, as recorded in Table 4.

The best fit kinetic model with regard to the experimental results of kerosene adsorption was the pseudo-second order model. This is indicated by the high values of their linear regressions, namely R^2^ > 0.98, for all samples as given in Table 4. However, the values of *q_e cal_* for the V/MWCNTs and V:Ce/MWCNTs were higher than the experimental values obtained, *q_t exp_*. The pseudo-second order model has been applied in the sorption of oil and metal ions over MWCNTs [65].

The third kinetic model (that is, intra-particle diffusion based on the theory proposed by Weber and Morris) was used to identify the diffusion mechanism [36]. The initial rate of intra-particle diffusion is expressed by Equation (9) [64]:

(9) $$q_{t}=K_{d}t^{1/2}+I$$

where *K_d_* denotes the intra-particle diffusion rate constant (mg/g min^1/2^) and I represents the intercept.

**Figure 12:**
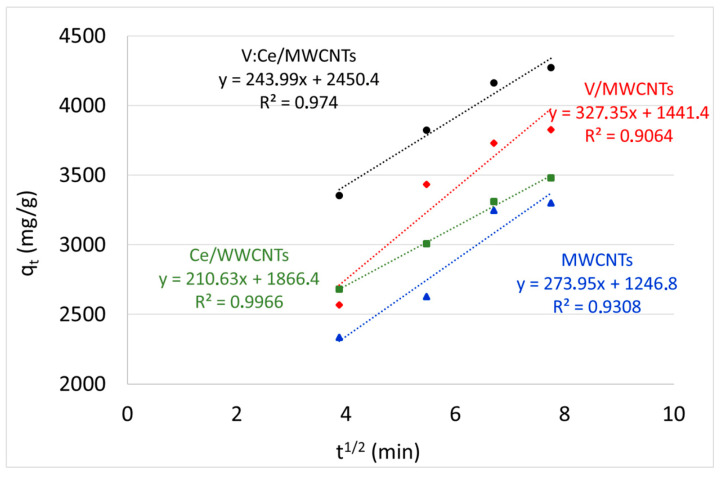
Intra-particle diffusion plot with regard to kerosene adsorption for metal oxide-modified MWCNTs.

According to this theory, the adsorbate uptake, *q_t_*, varies almost proportionally to the square root of the contact time, *t*^½^, rather than *t* [66]. Figure 12 is the outcome of this third kinetic model. The constant *K_d_* was obtained from the gradient of the plot of *q_t_* versus *t*^1/2^ (Table 4). The achieved plot does not pass through the origin with linear response values and R^2^ varied between 0.90 and 0.99 for all samples.

The intercept of the plot indicated the boundary layer effect during sorption [64]. Intra-particle diffusion would be considered the rate-limiting step if the plotted curve passed through the origin. Since the intercept (I) and intra-particle diffusion rate constant (*K_d_*) for all metal oxide-modified MWCNTs samples was large (Table 4), the surface sorption of kerosene is the rate-limiting step rather than intra-particle diffusion in the reported study.

**Figure 13:**
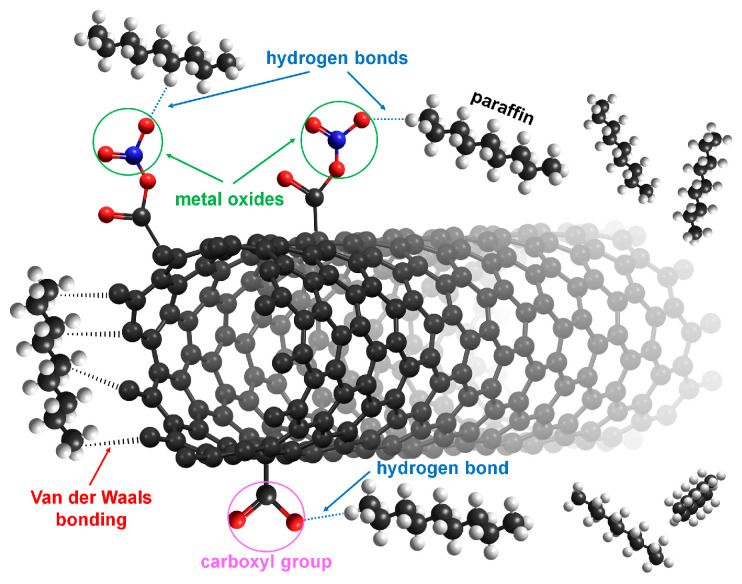
Schematic interaction/adsorption of the alkane molecules on the surface of MWCNTs samples.

Figure 13 shows schematic sorption of the alkane molecules on the surface of MWCNTs samples. One of the most probable ways of bringing about the sorption of nonpolar, alkane molecules on MWCNTs is via CH···π interactions. The CH···π link is one of the weak non-covalent hydrogen bonds. In the case of fresh MWCNTs, an interaction occurs between the hydrogen atoms of the saturated hydrocarbons (kerosene) and the carbon atoms of the MWCNTs. Also van der Waals interaction could take place. In the case of metal oxide-modified nanoparticles over MWCNTs, hydrogen bonding forms between the oxygen atoms in the metal oxide-modified nanoparticles over MWCNTs and the hydrogen atoms in kerosene [29,35].

## 4. Conclusions

The result showed that the novel MWCNTs based adsorbents are nanosized materials having major crystalline phase with regard to the graphene layers of the MWCNTs. The EDX and TGA studies confirmed the successful deposition/attachment of metal oxides onto MWCNTs surface. The deposition of V_2_O_5_ (2–4 wt%), CeO_2_ (7–8 wt%) and V_2_O_5_:CeO_2_ (8–12 wt%) oxides over the oxidized MWCNTs caused the blockage of micropores. At the same time the surface area remained relatively high (130–135 m^2^/g) for adsorption treatment.

The experimental results showed that adsorption capacity and removal efficiency of MWCNTs for kerosene removal from water increased after adding metal oxides nanocomposites over MWCNTs from 3300 to 4270 mg/g and 66 to 85%, respectively.

The obtained results were further analyzed through kinetic models. These results demonstrated that the best fit of experimental data was to the pseudo-second order model. Furthermore, the intra-particle diffusion kinetic model showed the presence of the boundary layer effect, thereby confirming the significant contribution of the rate-limiting step on the surface sorption of kerosene. Hydrocarbon attachment to adsorbent surfaces likely occurs mainly though the formation of weak hydrogen bonds.

The studied metal oxides modified MWCNTs can be considered as potential adsorbents for hydrocarbons (kerosene, oil etc.) depollution control and could open new avenues for their application.

## Figures and Tables

**Table 1:** Composition of metal oxide-modified MWCNTs.

Sample	Theoretical wt%	wt% by EDX
V/MWCNTs	2.0/98.0	2.3/97.7
Ce/MWCNTs	2.0/98.0	7.4/92.6
V:Ce/MWCNTs	6.0:2.0/92.0	6.0:2.1/91.9

**Table 2:** Weight loss during outgassing; total and micropore surface area, S_BET_ and S_micro_; volume of pores between 1.7 and 300 nm diameter and micropore volume, V_1.7–300 nm_ and V_micro_; average pore size, D_av_ values of fresh MWCNTs, acid-treated MWCNTs and metal oxide-modified MWCNTs.

Samples	Weight Loss (wt%)	S_BET_ (m^2^/g)	S_micro_ (m^2^/g)	V_1.7–300 nm_ (cm^3^/g)	V_micro_ (cm^3^/g)	D_av_ (nm)
MWCNTs	0.1	156	25.4	0.6577	0.0109	16.1
MWCNTs ox.	7.9	140	13.6	1.0384	0.0052	28.2
V_2_O_5_	0.8	3.0	0	0.0073	0	13.4
CeO_2_	0.5	69.4	15.5	0.1676	0.0069	13.4
V_2_O_5_:CeO_2_	3.5	8.8	0.5	0.0368	0.0001	15.0
V/MWCNTs	1.3	135	6.9	0.8439	0.0018	24.5
Ce/MWCNTs	4.5	115	0	0.7002	0	24.1
V:Ce/MWCNTs	6.9	129	0	0.8545	0	25.8

**Table 3:** GC results of the adsorption capacity and removal efficiency of kerosene from water, using fresh MWCNTs, Ce/MWCNTs, V/MWCNTs, and V:Ce/MWCNTs at different adsorption times.

MWCNTs			Ce/MWCNTs		V/MWCNTs		V:Ce/MWCNTs	
Time (min)	*RE* (%)	*q_t_* (mg/g)	*RE* (%)	*q_t_* (mg/g)	*RE* (%)	*q_t_* (mg/g)	*RE* %	*q_t_* (mg/g)
15	47	2335	54	2681	51	2567	67	3355
30	53	2626	60	3009	69	3435	76	3822
45	65	3247	66	3308	75	3731	83	4161
60	66	3300	70	3481	77	3825	85	4271

**Table 4:** Parameters of the applied kinetic model equations with regard to kerosene adsorption from the aqueous solution onto the samples studied.

Kinetic Models	Parameters	MWCNTs	Ce/MWCNTs	V/MWCNTs	V:Ce/MWCNTs
	*q_t exp_* (mg/g)	3300	3481	3825	4271
pseudo-first order	*k*_1_ (min^−1^)	0.089	0.061	0.090	0.090
	*q_e cal_* (mg/g)	4946	2394	5164	4674
	R^2^	0.8969	0.9631	0.9962	0.9494
pseudo-second order	*k*_2_ (g/mg min)	0.30 × 10^−4^	0.45 × 10^−4^	0.16 × 10^−4^	0.28 × 10^−4^
	*q_e cal_* (mg/g)	3333	3333	5000	5000
	R^2^	0.9847	0.9978	0.9975	0.9994
intra-particle diffusion	*K_d_* (mg/g min^1/2^)	274	2104	3274	244
	I	1247	1866	1441	2450
	R^2^	0.9308	0.9966	0.9064	0.9740

## Supplementary Materials

The following are available online at https://www.mdpi.com/article/10.3390/nano12020189/s1, Table S1: Spectral analysis data from the deconvolution of D- and G-bands and the calculated ratios. Ratio changes compared to the MWCNTs sample are displayed in brackets, Table S2: Mass loss data from the termoanalitical measurements, Figure S1: Change in kerosene concentration against time over MWCNTs, Ce/MWCNTs, V/MWCNTs and V:Ce/MWCNTs (*C*_0_ = 500 mg, V_sample_ = 0.05 L, m_ads_ = 0.005 g), Figure S2: Pseudo-first order plot for kerosene adsorption onto metal oxide-modified MWCNTs.
