# Supplementary material for: V2O5, CeO2 and Their MWCNTs Nanocomposites Modified for the Removal of Kerosene from Water
Source: Nanomaterials (Basel). 2022 Jan 6;12(2):189. doi: 10.3390/nano12020189 (PMC8778115; doi:10.3390/nano12020189)
Supplement: Supplementary file 1 [file nanomaterials-12-00189-s001.zip › nanomaterials-1482476-supplementary.pdf]

Table S1: Spectral analysis data from the deconvolution of D- and G-bands and the calculated ratios. Ratio changes compared to the MWCNTs sample are displayed in brackets.

Sample	Center	FWHM	Area	Intensity	I_D/I_G	$I_D/I_{G'}$	$I_{G'}/I_D$	A_D/A_G	$A_D/A_{G'}$	$A_{G'}/A_D$
MWCNTs	1284	71.130	0.04722	0.001782	1.48	1.84	0.54	1.59	1.05	0.96
	1289	36.760	0.05887							
	1598	36.995	0.06684	0.001207						
	2561	71.375	0.10150	0.000862						
MWCNTs ox.	1289	43.178	0.06766	0.001419	1.44 (-0.04)	1.65 (-0.19)	0.61 (0.06)	1.48 (-0.11)	0.97 (-0.08)	1.03 (0.08)
	1289	59.332	0.03089							
	1599	44.993	0.06651	0.000988						
	2567	76.308	0.10176	0.000862						
Ce/MWCNTs	1289	67.900	0.06237	0.002172	1.60 (0.12)	2.74 (0.89)	0.37 (-0.18)	1.76 (0.17)	2.44 (1.39)	0.41 (-0.55)
	1289	40.887	0.07774							
	1599	47.349	0.07962	0.001361						
	2567	59.131	0.05748	0.000794						
V/MWCNTs	1289	73.096	0.04876	0.001454	1.40 (-0.08)	2.30 (0.46)	0.43 (-0.11)	1.40 (-0.18)	2.08 (1.03)	0.48 (-0.48)
	1289	55.547	0.07993							
	1600	57.770	0.09178	0.001041						
	2561	67.739	0.06200	0.000632						
CV:Ce/ MWCNTs	1289	64.762	0.06845	0.002304	1.78 (0.31)	2.65 (0.81)	0.38 (-0.17)	2.42 (0.84)	2.24 (1.19)	0.45 (-0.51)
	1289	42.833	0.09338							

	1598	45.096	0.06680	0.001291						
	2566	60.566	0.07237	0.000868						

Table S2: Mass loss data from the termoanalitical measurements

Sample	T start (°C)	T end (°C)	Mass loss step	Total mass loss	Residual mass	Estimated Ce-/V-oxide content (m/m%)
MWCNTs	21	190	0.15 mg (2.5 %)	5.28 mg (89.6 %)	0.614 mg (10.4 %)	n/a
	190	440	0.05 mg (0.8 %)			
	440	750	5.08 mg (86.2 %)			
	750	1016	0 mg (0 %)			
MWCNTs ox.	21	155	1.43 mg (20.1 %)	7.02 mg (98.5 %)	0.108 mg (1.5 %)	n/a
	155	366	1.32 mg (18.5 %)			
	366	500	0.32 mg (4.5 %)			
	500	730	3.84 mg (53.9 %)			
	730	1016	0.11 mg (1.5 %)			
Ce/MWCNTs	22	190	0.35 mg (5.1 %)	6.04 mg (86.6 %)	0.938 mg (13.4 %)	CeO_2: 11.9%
	190	360	0.33 mg (4.7 %)			
	360	485	0.49 mg (7 %)			
	485	700	4.86 mg (69.6 %)			

	700	1015	0.01 mg (0.2 %)			
V/MWCNTs	22	190	0.58 mg (8.4 %)	6.6 mg (94.8 %)	0.364 mg (5.2 %)	V_2O_5: 3.7%
	190	330	0.4 mg (5.7 %)			
	330	418	0.88 mg (12.6 %)			
	418	590	4.59 mg (65.8 %)			
	590	700	0.15 mg (2.1 %)			
	700	1015	0.01 mg (0.2 %)			
V:Ce/MWCNTs	21	190	0.52 mg (7.4 %)	6.21 mg (90.0 %)	0.694 mg (10.0 %)	CeO_2+V_2O_5: 8.5%
	190	316	0.25 mg (3.6 %)			
	316	422	1.02 mg (14.6 %)			
	422	545	3.42 mg (49.1 %)			
	545	700	1.01 mg (14.6 %)			
	700	1016	0.06 mg (0.8 %)			

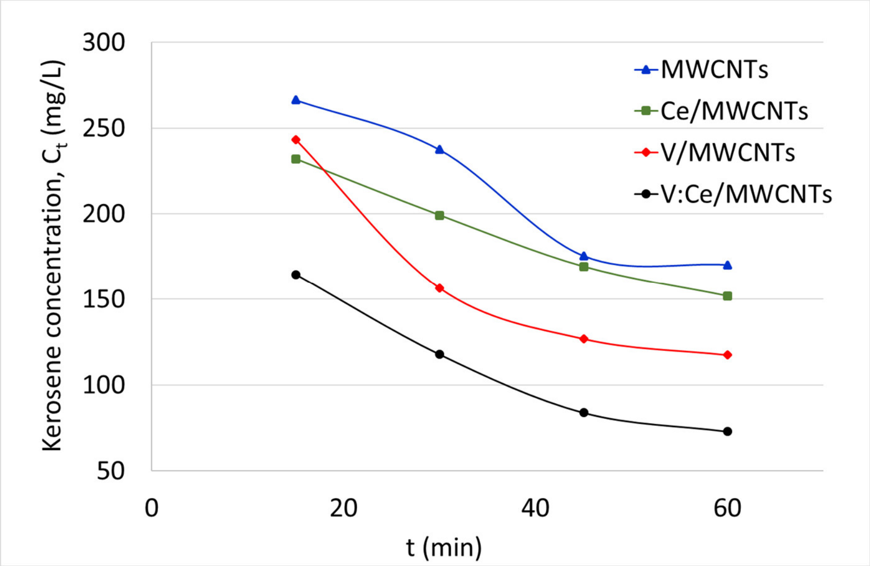

Figure S1. Change in kerosene concentration against time over MWCNTs, Ce/MWCNTs, V/MWCNTs and V:Ce/MWCNTs (C_0 = 500 mg, V_{sample} = 0.05 L, m_{ads} = 0.005 g).

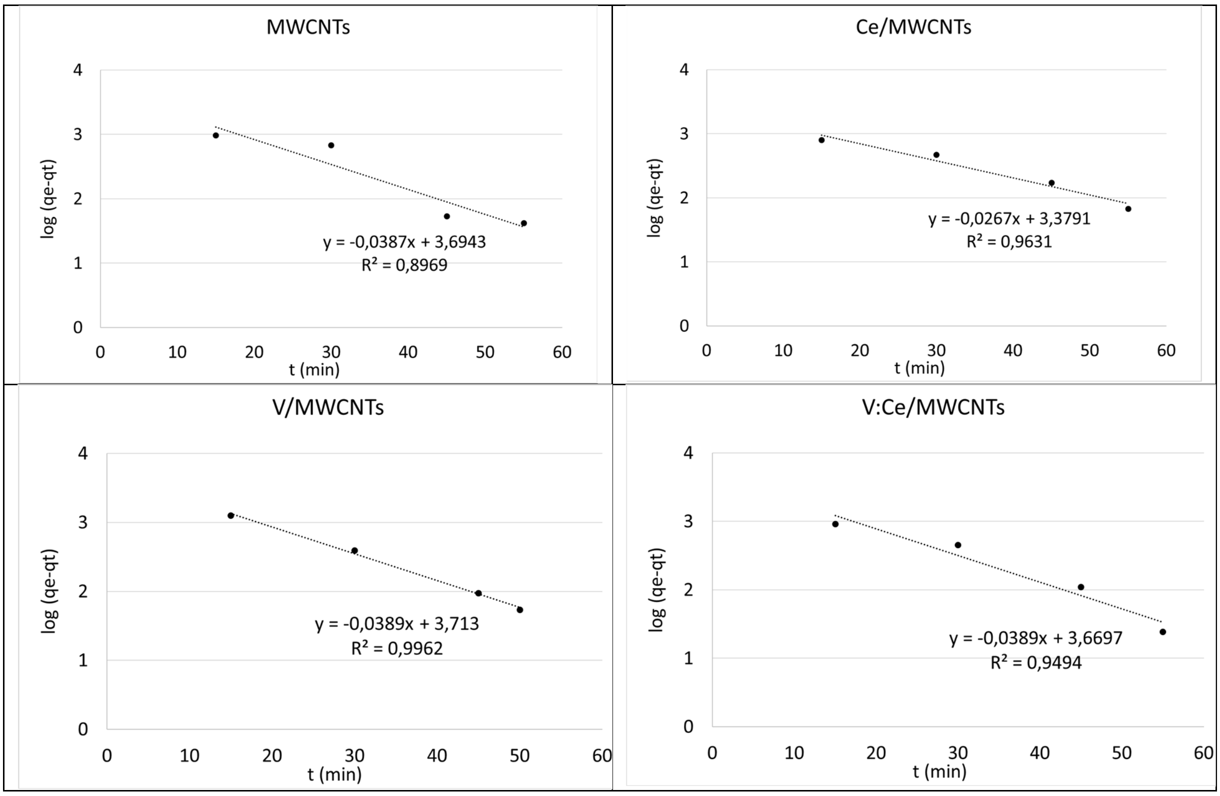

Figure S2. Pseudo-first order plot for kerosene adsorption onto metal oxide-modified MWCNTs.
